# BHLHE40 and ChREBP associate with hepatic enhancer clusters containing PPARα, RXRα, and HNF4 nuclear receptors

**DOI:** 10.1210/endocr/bqag095

**Published:** 2026-08-21

**Authors:** Krista Kokki, Sylvia J Wowro, Marius Robciuc, Atte Hallasaari, Linda Porri, Matias Kinnunen, Catrin A Stegmann, Konstantin M Petricek, Markku Varjosalo, Michael Schupp, Ville Hietakangas

**Affiliations:** Faculty of Biological and Environmental Sciences, University of Helsinki, 00790 Helsinki, Finland; Institute of Biotechnology, Helsinki Institute of Life Science, University of Helsinki, 00790 Helsinki, Finland; Max Rubner Center for Cardiovascular Metabolic Renal Research (MRC), Charité-Universitätsmedizin Berlin, corporate Member of Freie Universität Berlin, Humboldt-Universität zu Berlin, Institute of Pharmacology, 10115 Berlin, Germany; Faculty of Biological and Environmental Sciences, University of Helsinki, 00790 Helsinki, Finland; Institute of Biotechnology, Helsinki Institute of Life Science, University of Helsinki, 00790 Helsinki, Finland; Faculty of Biological and Environmental Sciences, University of Helsinki, 00790 Helsinki, Finland; Institute of Biotechnology, Helsinki Institute of Life Science, University of Helsinki, 00790 Helsinki, Finland; Faculty of Biological and Environmental Sciences, University of Helsinki, 00790 Helsinki, Finland; Institute of Biotechnology, Helsinki Institute of Life Science, University of Helsinki, 00790 Helsinki, Finland; Institute of Biotechnology, Helsinki Institute of Life Science, University of Helsinki, 00790 Helsinki, Finland; Max Rubner Center for Cardiovascular Metabolic Renal Research (MRC), Charité-Universitätsmedizin Berlin, corporate Member of Freie Universität Berlin, Humboldt-Universität zu Berlin, Institute of Pharmacology, 10115 Berlin, Germany; Max Rubner Center for Cardiovascular Metabolic Renal Research (MRC), Charité-Universitätsmedizin Berlin, corporate Member of Freie Universität Berlin, Humboldt-Universität zu Berlin, Institute of Pharmacology, 10115 Berlin, Germany; Institute of Biotechnology, Helsinki Institute of Life Science, University of Helsinki, 00790 Helsinki, Finland; Max Rubner Center for Cardiovascular Metabolic Renal Research (MRC), Charité-Universitätsmedizin Berlin, corporate Member of Freie Universität Berlin, Humboldt-Universität zu Berlin, Institute of Pharmacology, 10115 Berlin, Germany; Faculty of Biological and Environmental Sciences, University of Helsinki, 00790 Helsinki, Finland; Institute of Biotechnology, Helsinki Institute of Life Science, University of Helsinki, 00790 Helsinki, Finland

**Keywords:** BHLHE40/DEC1, ChREBP, sugar, hepatocyte, ChIP-seq, RNA-seq

## Abstract

BHLHE40/DEC1 is a basic helix-loop-helix transcription factor (TF) that regulates circadian rhythm and T-cell responses. In hepatocytes, its function and interplay with other TFs are poorly understood. Employing a genome-wide approach, we show that its genomic binding strongly overlapped with that of carbohydrate response-element binding protein, a sugar-sensing TF and known inducer of BHLHE40 expression. Transcriptomic analysis of primary mouse hepatocytes revealed reduced expression of genes involved in genomic stability on *Bhlhe40* knockdown by siRNA. *Bhlhe40* depletion potentiated fructose responsiveness of genes involved in cell-cycle regulation. Strikingly, genomic binding of BHLHE40 extensively overlapped with enhancers occupied by PPARα, RXRα, and HNF4 nuclear receptors and BHLHE40 fine-tuned the expression of PPARα target genes. Using HEK293 cells, we further observed that BHLHE40 physically interacted with RXRα and PPARα cofactors. Collectively, our data suggest that through cooperation with carbohydrate response-element binding protein and nuclear receptors, BHLHE40 is a central regulator of hepatic gene expression with potential to integrate inputs from nutrient signals contributing to the metabolic flexibility of the liver.

Liver plays a central role in maintaining energy homeostasis by overseeing key biochemical pathways involved in lipid, carbohydrate, and protein metabolism, as well as bile synthesis and detoxification of drugs and toxins. To achieve these functions, pioneer transcription factors (TFs) such as FOXA and HNF4α define the liver genetic profile (1, 2), whereas core TFs such as PPARs and carbohydrate response-element binding protein (ChREBP) integrate the environmental cues to control lipid and carbohydrate metabolism, and energy homeostasis (3, 4). Cross-talk between PPARα and ChREBP was shown to promote the glucose-induced FGF21 expression, a hepatokine that contributes to inter-tissue coordination of energy homeostasis (5). Recent evidence from multi-omics studies showed that an extended array of TFs beyond the core transcriptional regulatory circuitry define the hepatocyte cell identity and function (6). Defining the roles of these TF interactions in controlling gene expression is necessary for understanding the regulation of hepatic functions in specific conditions.

Basic Helix-Loop-Helix Family Member E40 (BHLHE40), also known as DEC1, SHARP2, or STRA13, is a transcription factor involved in a wide array of biological processes, including circadian rhythm (7), cellular differentiation, cell growth, immune responses, and cancer (8, 9). While its repressive roles in the circadian clock are known to be conserved in *Drosophila* and mammals, other functions such as repression of lipogenesis have only been observed in mammals (10, 11). In mice, BHLHE40 deficiency enhances brown adipose tissue lipolysis and prevents high-fat diet-induced obesity (12). In the liver, it is regulated by circadian rhythm, fasting and LXR nuclear receptors, and it is required for insulin induction of SREBP-1c (13-16).

BHLHE40 is a downstream target of ChREBP and it has been suggested to act as a negative feedback regulator of ChREBP target genes *Fasn* and *Pklr* through binding of same regulatory regions (10). In this study, we analyzed the interplay between BHLHE40 and ChREBP by comparative analysis of their genomic targets and analyzing the contribution of BHLHE40 to glucose and fructose-induced gene expression in mouse hepatocytes. Our data show that the genomic binding of BHLHE40 and ChREBP display pervasive, but not complete, overlap and that the role of BHLHE40 in modulating the sugar responsive transcriptional output is more complex than previously anticipated. Surprisingly, our in silico motif enrichment and comparative chromatin immunoprecipitation sequencing (ChIP-seq) data analyses identified that the genomic sites of BHLHE40 and ChREBP were also broadly occupied by hepatic nuclear receptors HNF4α, RXRα, and PPARα. This was consistent with the results of unbiased proteomic analysis of BHLHE40-proximal proteins, uncovering interactions with RXR and PPARα cofactors. Subsequent functional analyses on gene regulation further revealed that BHLHE40 is necessary for full activation of PPARα-responsive genes. Collectively, our data suggest that BHLHE40 belongs among the core transcriptional regulators of hepatocytes, acting on joint genomic targets together with other well-established transcriptional regulators of liver metabolism.

## Materials and methods

### Animal experimentation

Animal procedures and primary hepatocyte isolation were approved by the corresponding authorities in Berlin/Germany (application G 0130-17, G0076-22, O 0400-17).

### Immunofluorescence

Liver samples were collected from C57BL/6JRccHsd male mice fasted for 7 hours during the light cycle and fixed with 4% paraformaldehyde in PBS, overnight (O/N) in +4 °C. Liver pieces were embedded in 2% agar and cut in 100- or 200-μm sections with a vibratome. Sections were blocked and permeabilized with staining buffer (PBS containing 5% normal goat serum, 0.3% TritonX-100, and 0.05% sodium azide) for 1 hour at room temperature (RT) and stained for BHLHE40/Dec1 using Novus Bio antibody (Novus Cat# NB100-1800, RRID:AB_10000524), diluted 1:100 in staining buffer and incubated O/N at RT. Sections were washed 4 to 5 times at RT, for 5 to 6 hours, and incubated with secondary antibody (Invitrogen/Molecular Probes Cat# A-11008, RRID:AB_143165) and phalloidin-Alexa568 (Invitrogen, cat # A12380), O/N at RT, followed by the same washing steps, DAPI staining and mounting using a 60- or 120-μm spacer. Images were acquired using Leica SP8 laser confocal microscope and processed using Fiji/ImageJ.

### Western blot

Livers of ad libitum-fed or 24 hours fasted mice were homogenized and total protein isolated by standard methods and separated by SDS-PAGE as described previously (17). Primary antibodies were used as following: ChREBP (Novus Cat# NB400-135F, RRID:AB_3188362, 1:1000 dilution), BHLHE40 (Novus Cat# NB100-1800, RRID:AB_10000524, 1:333 dilution), and GAPDH (Cell Signaling Technology Cat# 2118, RRID:AB_561053, 1:1000 dilution). A secondary antibody conjugated to horseradish peroxidase (Pierce) and a chemiluminescent kit was used for detection (ThermoFisher). Uncropped western blot images are provided in the supplementary file 3 deposited in (18).

### Quantitative PCR analyses

Total RNA from liver tissue or primary hepatocytes was isolated using standard spin column kits (VWR). cDNA synthesis was performed using M-MLV reverse transcriptase (Promega) and quantitation was carried out by Sybr green quantitative PCR (qPCR; Eurogentec) using standard curves for each gene, normalized to the expression of *Rplp0* as housekeeping gene. Primer sequences are listed in the Supplementary file 4 deposited in (18).

### ChIP-seq and related analysis

BHLHE40 and ChREBP data were generated in-house and previously reported (GSE207198) (11). Small pieces of frozen liver tissue from ad libitum-fed male mice (JAX:000664 obtained from the Jackson Laboratory) were pulverized in liquid nitrogen and cross-linked in 1% formaldehyde for 15 minutes, followed by quenching with 1/20 volume of 2.5 M glycine solution, and 1 wash with 1× PBS. Nuclear extracts were prepared by homogenizing in 20 mM HEPES, 0.25 M sucrose, 3 mM MgCl_2_, 0.2% NP-40, and 3 mM β-mercaptoethanol; complete protease inhibitor tablet (Roche). Chromatin fragmentation was performed by sonication in 50 mM HEPES, 1% SDS, and 10 mM EDTA, using a Bioruptor (Diagenode) for 20 cycles of 30 seconds at the highest level. Proteins were immunoprecipitated in 50 mM HEPES/NaOH at pH 7.5, 155 mM NaCl, 1.1% Triton X-100, 0.11% Na-deoxycholate, and complete protease inhibitor tablet, using anti-ChREBP (Novus Cat# NB400-135F, RRID:AB_3188362) or anti-BHLHE40 (Novus Cat# NB100-1800, RRID:AB_10000524) antibodies. Antibodies were precipitated with a 33% slurry of precleaned protein A Sepharose beads in 0.5% BSA/PBS for 2 hours. Cross-linking was reversed overnight at 65 °C and DNA isolated using phenol/chloroform/isoamyl alcohol. ChIP-seq was performed on DNA of 2 individual C57Bl/6 mice, released from the respective antibody (2× BHLHE40, 2× ChREBP, and 2× input control) with an average depth of 20 million reads per sample, 75-bp single-end reads at the DNA Genomics and Sequencing core facility at the University of Helsinki, Finland. The samples were run on the Illumina NextSeq500 using the 75-bp cycle kit (Illumina). Raw ChIP-seq data for *Mus musculus* liver RXR (GSE35262) (19), PPARα (GSE61817) (20), and HNF4α (GSE90533) (21) were obtained from Gene Expression Omnibus. Samples were mapped to the genome (*Mus musculus*: NCBI GRCm38) with BWA (v.0.7.17), reads below quality 5 were discarded, and maximum mismatches in seed were set to 2 with first 32 subsequences as seed. Unmapped, nonprimary and alignments below mapping quality 30 were discarded along duplicates using samtools (v.1.4) and picard (v2.18.10). Fragment sizes were estimated with R/Bioconductor phantompeaktools (v.1.2.2) (22). Peaks with q-values < 0.05, derived from dynamic Poisson model and Benjamini-Hochberg correction, were called for primary chromosomes with MACS2 (v.2.1.0). Empirical blacklist for mm10 was obtained from ENCODE (https://www.encodeproject.org). Peaks in all replicates were defined as expressed and annotated to the nearest TSS from the middle of the peak using the respective genome (*Mus musculus*: NCBI GRCm38) with R/Bioconductor's ChIPpeakAnno (v.3.18.2) package (23, 24). For visualization, the binding profiles were merged using samtools merge. Heatmaps of binding profiles for merged BHLHE40 and ChREBP were created with deepTools (v.3.5.1) using their respective reference points (25). Overlapping peaks between BHLHE40 and ChREBP were created using bedtools (v.2.29.2) intersect with a minimum fraction of 50% in both conditions (26), whereas R/Bioconductor's UpSetR (v.1.4.0) was used for finding overlapping peaks between the 5 transcription factors (BHLHE40, ChREBP, PPARα, HNF4α, and RXR) with distinct mode (27). The peak visualizations were performed using by R/Bioconductor's karyoploteR (v.1.32.0) package (28).

### Primary hepatocyte culture and treatments

Primary hepatocyte isolation from male C57Bl6/J mice and siRNA-mediated knockdown were performed as previously described (29). Primary hepatocytes were infected with equal titers of GFP or *Bhlhe40*-expressing adenoviruses (Vector Biosystems, USA) overnight. The next day, cells were washed and incubated with 50 µM of WY-14643 or DMSO vehicle for another 24 hours.

### RNA-seq and related analysis

For RNA-seq, we used an 8 week-old male C57Bl6/J mouse to isolate the hepatocytes. Briefly, cells were plated in 12-well plates and transfected in high-glucose DMEM using Optimem, 1 Control siRNA and 2 siRNAs targeting *Bhlhe40*, with 4 to 6 wells for each experimental condition. Sequences of the used siRNA oligonucleotides are listed in supplementary file 4 deposited in (18). After a 4-hour incubation in low-glucose (2.5 mM) DMEM cells incubated for 24 hours in low-sugar media (LSM, 2.5 mM glucose DMEM), or DMEM with high glucose (27.5 mM) or high fructose (25 mM fructose and 2.5 mM glucose). All wells were analyzed by qPCR to test whether primary hepatocytes responded to low- and high-sugar concentrations with elevated Txnip expression as positive control, and whether Bhlhe40-targeting siRNA reduced its mRNA expression by more than 50%. Four wells from each experimental condition were used further as ex vivo experimental replicates in the downstream analysis. RNA was extracted using Nucleospin RNA II kit (Macherey-Nagel), ribosomal RNA depleted, and libraries were constructed with TruSeq Stranded mRNA Library Prep Kit (Illumina). Samples were sequenced using Illumina technology to an average depth of 40 M reads per sample. Each sample was sequenced twice in separate runs.

The RNA-seq data were processed using nf-core/rnaseq pipeline v.3.19.0 from the nf-core collection of workflows (30), using software environments from the Bioconda and Biocontainers projects. The pipeline was executed with Nextflow v.24.10.3. The 2 runs for each sample were pooled together. Data set was assessed with FastQC v.0.12.1 and fastp v.0.24.0 was used for read trimming. The first 9 bases from each read were trimmed to unify the quality and reads were required to have minimum phred quality score of 15. The reads were mapped to the Mus musculus reference genome GRCm39 with STAR v.2.7.11b and the mapping was quantified with Salmon v.1.10.3. The differential expression was quantified with DESeq2 package v.1.46.0 implemented in R v.4.4.2/Bioconductor v.3.20,with design formula containing siRNA treatment, diet group and their interaction term. Genes were required to have minimum of 10 reads in at least 4 samples to be considered in the analysis. Benjamini–Hochberg correction was used for adjusting *P* values. Genes with an adjusted *P* < .05 and absolute log2 fold change >0.5 were considered differentially expressed genes (DEGs). The raw sequencing data is available on Gene Expression Omnibus using the accession number GSE316769.

### Pathway enrichment analysis

ChipEnrich v.2.16.0 was used for identifying enriched gene sets on the ChIP-seq datasets (31). Nearest gene was used as gene locus definition and predefined mm10 was used as the genome basing the peaks file. The enrichment was performed from manually downloaded KEGG databases. Pathways with Benjamini–Hochberg adjusted *P* < .05 were considered significantly enriched. For RNAseq and interactome datasets, GSEA and overrepresentation analysis were performed using the clusterProfiler v.4.14.6 (32) and ReactomePA v.1.50.0 packages in R/Bioconductor using KEGG v.116.0 and Reactome v.94 databases. Pathways with an adjusted *P* value < .05 with Benjamini–Hochberg correction were considered significantly enriched. For GSEA, the gene sets from RNAseq data were ranked using ashr-shrunk log2 fold changes. For the clustering in the gene concept network plot, we retrieved the ancestral class for each enriched pathway from the Reactome database. The pathway overlaps were calculated based on core enrichment genes using Jaccard similarity. The results were plotted using ggraph v.2.2.2 in R.

### Motif enrichment analysis

MEME-ChIP v.4.12.0 was used for de novo motif discovery, comparison and identification with the motif databases provided by the MEME Suite for overlapping peaks between BHLHE40 and ChREBP (33, 34). Motifs with E-value < 0.05 were considered significantly similar as calculated using Tomtom software (35). The Markov background model was created by using MEME tools. FASTA sequences of 200-bp regions centered on ChIP-peak summits were fetched from *Mus musculus* (NCBI GRCm38) by using bedtools v.2.29.2 getfasta.

### Biotin proximity labeling analysis of protein-protein interactors

The biotin proximity labeling workflow was performed as previously detailed (36), with the following exceptions. In this article, the BHLHE40 was tagged with N-terminal MAC3-tag (37), which contains a smaller UltraID biotin ligase (38). The BHLHE40 cell line, generated from Flp-In T-REx 293 cells (Thermo Fisher Scientific) was induced at 70% confluency by adding 2 μg/mL tetracycline for 16 hours, after which 50 μM biotin was supplemented for 8 hours before harvesting duplicate samples with 6 × 10^7^ cells per sample. Samples were lysed in 1.5 mL volume and biotinylated proteins bound to 125 μL Strep-Tactin XT 4Flow resin (iba-lifesciences) and proteins eluted with 240 μL volume with 0.4 nM biotin 3 times. Eluted samples were trypsinized overnight and desalted using BioPureSPN MINI C18 columns (The nest Group, Inc.), after which samples were dried and subsequently dissolved into 30 μL volume of 0.1% TFA and 1% ACN in water and 3 μL of samples were loaded into an Evotip pure tips (Evosep Biosystems). Samples were analyzed on a TimsTOF pro mass spectrometer (Bruker) with a CaptiveSpray nano-electrospray ion source coupled to an Evosep 1 liquid chromatography system (Evosep Biosystems). Peptide separation was achieved using an 8 cm × 150 µm column packed with 1.5 µm C18 beads (EV1109, Evosep), which a 21-minute gradient time. Mobile phase A 0.1% formic acid in water and mobile phase B 0.1% formic acid in acetonitrile. MS analysis was conducted in positive-ion mode using a data-dependent acquisition approach in parallel accumulation serial fragmentation mode, using the data-dependent acquisition-parallel accumulation serial fragmentation-short_gradient_0.5s-cycletime method using a 20-minute gradient. Mass spectrometer result files were searched using Fragpipe v23.0 (https://fragpipe.nesvilab.org/). The detected sample proteins were scored against MAC3-tag GFP control samples using SAINT (39) and filtered by BFDR 0.01 to determine the high-confidence interactions.

### Data visualization

Graphs were generated in GraphPad Prism, InkScape v.1.4.2, or R-studio using packages ggplot2 v.4.0.0, ggvenn v.0.1.19, ComplexUpset v.1.3.3, UpSetR v.1.4.0, karyoploteR v.1.32.0, igraph v.2.1.4, and ggraph v.2.2.2.

## Results

### BHLHE40 binding displays extensive genomic overlap with ChREBP in hepatocytes

**Figure 1:**
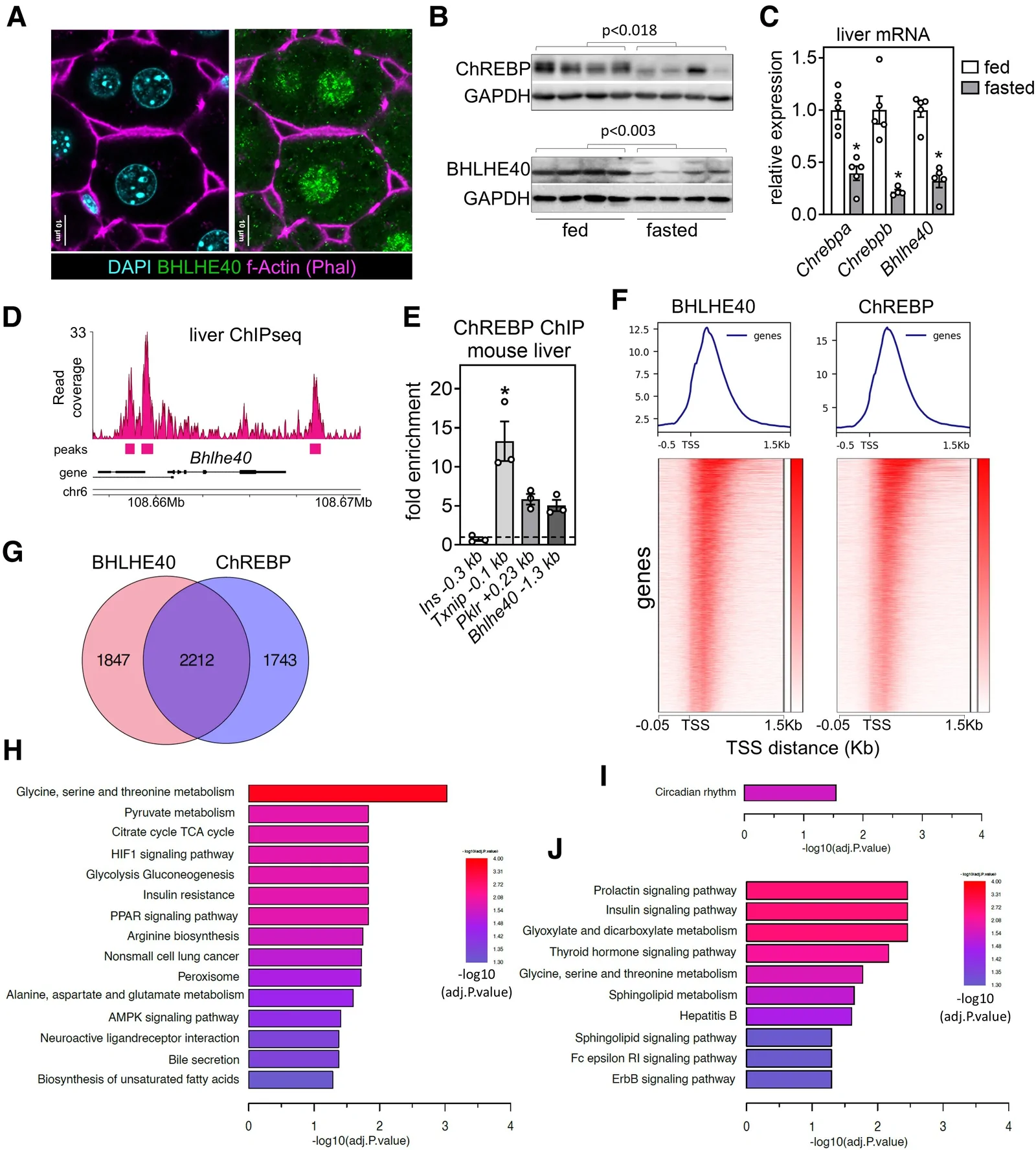
BHLHE40 and ChREBP show overlapping genomic binding in mouse liver. (A) Staining of BHLHE40 in liver of mice fasted for 7 hours. (B) Expression of hepatic ChREBP and BHLHE40 protein (n = 4) determined by immunoblotting and (C) mRNA expression (n = 5) of *Chrebpa* (*P = .0007), *Chrebpb* (*P = .0013), and *Bhlhe40* (*P < .0001) analyzed by qPCR in mice fed ad libitum or fasted for 24 hours. (D) ChREBP-binding profile near the *Bhlhe40* gene and (E) enrichment of ChREBP at genomic sites near its target genes (*Txnip* −0.1 kb *P = .0005) determined by ChIP qPCR (n = 3). (F) Heatmap and binding profile showing all peak regions from ChIP-seq enrichment for BHLHE40 and ChREBP in relation to the transcription start site (TSS) of bound genes. (G) Called peaks with >50% overlap between BHLHE40 and ChREBP datasets. (H) Pathway analysis (adj. *P* value < .05) of shared peaks between BHLHE40 and ChREBP (I) unique peaks for BHLHE40 and (J) unique peaks for ChREBP. In (C) and (E), data are shown as individual data points and mean ± sem. (D-J) Liver ChIP-seq of C57BL/6J male mice, n = 2 for ChREBP, BHLHE40, and input ctrl, respectively. Merged ChIP-seq binding profiles and called peak locations with q value < 0.05 were analyzed. *P < .05, unpaired 2-tailed Student *t*-test (B, C, and E) vs mice fed ad libitum (B and C) or vs a site unbound by ChREBP (*Ins* −0.3 kb) by 1-way ANOVA (E). Protein expression was normalized to GAPDH (B) and RNA to *Rplp0* (C).

First, we wanted study the expression and nutrient regulation of BHLHE40 in the liver. Staining of BHLHE40 in liver from mice fasted for 7 hours showed a constitutive nuclear localization (Fig. 1A). Prolonged fasting caused a significant reduction (*P* < .05, N = 4-5) at protein and mRNA levels (Fig. 1B and 1C) similar to that observed for ChREBP (10, 11, 14). To explore if BHLHE40 and ChREBP bind genomic regions close to similar gene sets in the liver, we performed ChIP-seq experiments in ad libitum-fed C57BL/6J male mice (n = 2 for ChREBP, BHLHE40, and input control, respectively). Merged ChIP-seq data were analyzed for called peak location with q-value < 0.05. We validated the sequencing results by qPCR for selected genes and observed that ChREBP binds directly to *Bhlhe40* promoter at comparable levels to established ChREBP targets such as *Txnip* and *Pklr* and that the ChIP qPCR robustly recapitulates the results obtained by ChIP-seq for both BHLHE40 and ChREBP (Fig. 1D and 1E and Fig. S1, deposited in (18)). Globally, BHLHE40 and ChREBP binding is strongly enriched in regions just downstream of transcription start sites (Fig. 1F) and have 54% overlap in targeted genes regardless of the stringent requirement of 50% overlap with each peak (Fig. 1G).

Pathway enrichment analysis (adj. *P* < .05) for significantly called peaks (q < 0.05) revealed that common targets of BHLHE40 and ChREBP are part of several metabolic pathways, such as amino acid metabolism, TCA cycle as well as peroxisomal genes and regulatory pathways, such as AMPK and PPAR signaling pathways, in addition to the established lipogenic genes (Fig. 1H). The genes bound by BHLHE40, but not ChREBP, are connected to circadian rhythm (Fig. 1I), whereas the genes bound by ChREBP, but not BHLHE40, are connected to cellular signaling, for example pathways controlled by insulin, prolactin, and thyroid hormone (Fig. 1H). These processes are highlighted by the common target genes *Pten*, a negative regulator of PI3K/AKT signaling; and *Mlxipl*, the ChREBP encoding gene (Fig. S2A, deposited in (18)). Examples of BHLHE40 specific target genes are *Clock* and *Nr1d1* (Fig. S2B, deposited in (18)), and *Akt1* and *mTor* for ChREBP (Fig. S2C, deposited in (18)). Collectively, these data indicate that BHLHE40 and ChREBP target a highly overlapping set of genes in hepatocytes.

### Transcriptomic effects of BHLHE40 knock down on sugar-responsive gene expression

**Figure 2:**
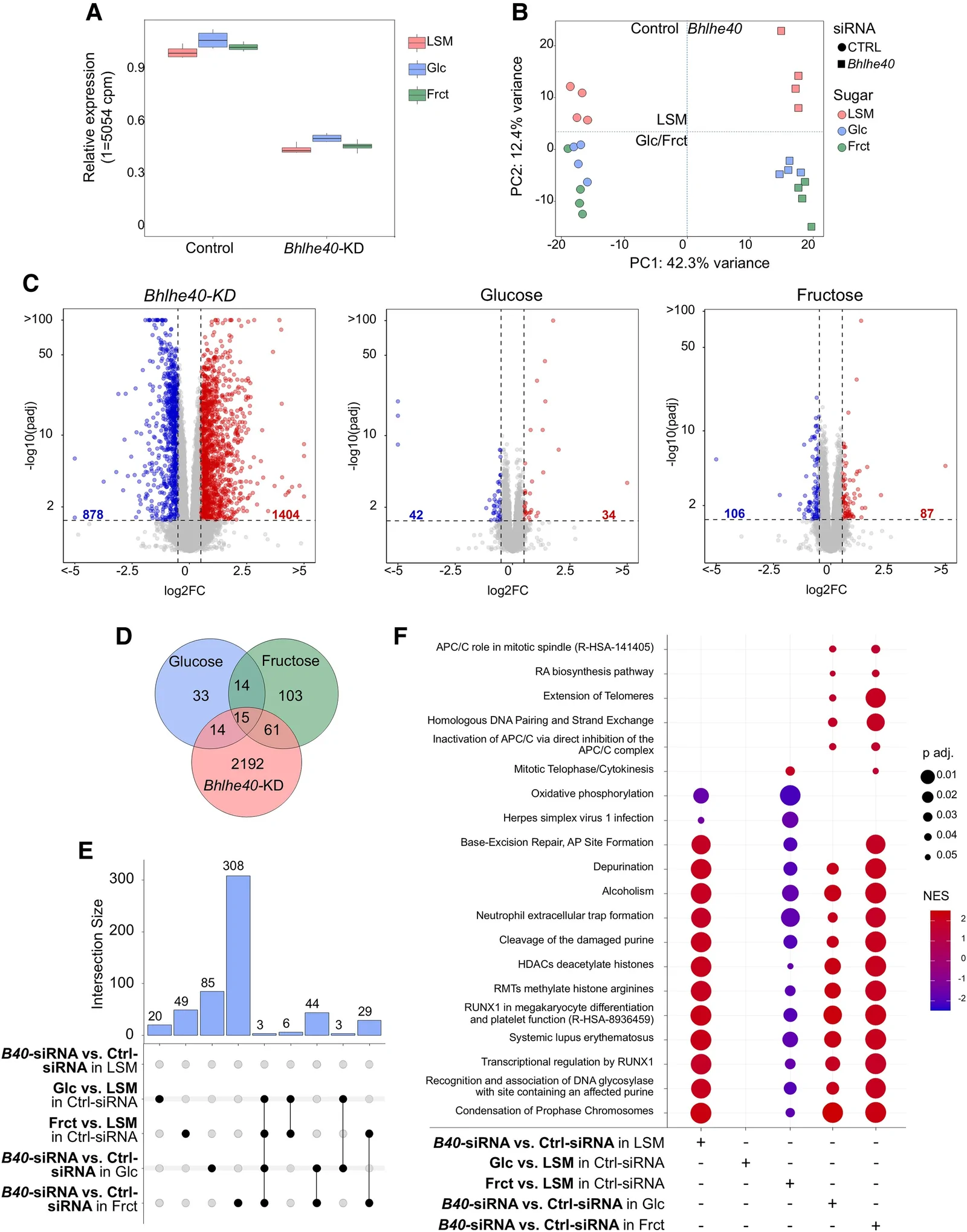
BHLHE40 depletion and ChREBP activation control distinct gene expression programs in primary hepatocytes. Control and *Bhlhe40*-KD mouse hepatocytes were incubated with low-sugar medium (LSM), high glucose (Glc), or high fructose (Frct) (n = 4, all groups). (A) *Bhlhe40* mRNA levels. (B) Principal component analysis (PCA). (C) Volcano plots highlighting the significantly downregulated (blue) and upregulated (red) genes (log2FC < −0.5 or >0.5, respectively, *P*. adj < .05) by the indicated conditions: *Bhlhe40-KD* (*Bhlhe40-*siRNA vs Ctrl-siRNA in LSM), Glc (Glc vs LSM in Ctrl-siRNA), and Frct (Frct vs LSM in Ctrl-siRNA). (D) Venn diagram of DEGs depicted in panel (C). (E) Number of shared genes that are differentially expressed upon Glc and Frct treatments in Control-siRNA (high ChREBP activation) or on *Bhlhe40*-siRNA treatment in cell with high ChREBP activity, excluding the DEGs caused by *Bhlhe40*-KD only (*B40*-siRNA vs Ctrl-siRNA in LSM). (F) Enriched pathways (adj. *P* < .05) that are common between the indicated groups. Pathway enrichment was obtained by gene set enrichment analysis (GSEA). For E and F, bold fonts indicate the experimental conditions for calculating DEGs and regular fonts the background condition.

To further explore the role of BHLHE40 in gene regulation we performed transcriptomic analysis of primary mouse hepatocytes in which BHLHE40 was depleted by siRNA (*Bhlhe40*-KD). We cultured both control and *Bhlhe40*-KD hepatocytes in low-sugar media (LSM) or high-glucose/fructose media, which cause either low or high ChREBP activity, respectively. RNA-seq analysis showed a robust 50% to 60% reduction of *Bhlhe40* mRNA levels in all culturing conditions (Fig. 2A). Principal component analysis indicated that samples cluster primarily according to the expression levels of *Bhlhe40*, but also moderately with the sugar exposure in the culture media (Fig. 2B). Accordingly, we detected 2282 differentially expressed genes (DEGs, log2FC < −0.5 or >0.5, adj. *P* < .05) in *Bhlhe40*-KD cells (*Bhlhe40-*siRNA vs Ctrl-siRNA in LSM) and only 240 DEGs in cells exposed to high glucose and high fructose combined (high glucose or high fructose vs LSM in Ctrl-siRNA) (Fig. 2C and 2D). *Bhlhe40-*KD caused 1.6-fold more genes to display elevated mRNA levels than reduced mRNA levels (Fig. 2C), in line with the reported role of BHLHE40 as transcriptional repressor (7, 8, 40). Of the genes that responded to glucose and fructose (ChREBP activation), only 90 of the 240 combined DEGs were also affected by *Bhlhe40-*KD (Fig. 2D).

Next, we analyzed the shared DEGs between cells with high ChREBP activity only (high glucose or high fructose vs LSM in Ctrl-siRNA) and cells with high ChREBP activity and BHLHE40 depletion (*Bhlhe40-*siRNA vs Ctrl-siRNA in high glucose or high fructose) (Fig. 2E). We found that most DEGs were distinct rather than common between these experimental conditions. Furthermore, cells with high ChREBP activity and BHLHE40 depletion had much higher number of DEGs compared to cells with high ChREBP activity only (Fig. 2E). In sum, these data suggest a much broader role of BHLHE40 in hepatocyte gene regulation than previously recognized. Notably, most *Bhlhe40*-KD responsive genes are not affected by sugar exposure.

To understand the cellular processes that are influenced by *Bhlhe40*-KD we performed GSEA with a focus on pathways that are connected to high ChREBP activity (Fig. 2F) and those that are independent of sugar levels in the culture media (Fig. S3A, deposited in (18)). As depicted in Fig. 2C, *Bhlhe40-*KD (*Bhlhe40-*siRNA vs Ctrl-siRNA in LSM) resulted in a larger number of genes to be upregulated than downregulated causing almost all pathways that were significantly enriched (adj. *P* < .05) to have a positive Normalized Enrichment Score (Fig. 2F). For ChREBP activation, only high-fructose condition (Frct vs LSM in Ctrl-siRNA) resulted in significantly enriched pathways (adj. *P* < .05), possibly because of its more potent effect on hepatocytes than high glucose (Fig. 2C-2E). The *Bhlhe40*-KD dominant effect was also observed in the GSEA, where all the downregulated pathways in cells treated with high fructose alone (Frct vs LSM in Ctrl-siRNA) was upregulated because of *Bhlhe40*-KD (*Bhlhe40-*siRNA vs Ctrl-siRNA in Frct). Largely, these upregulated pathways affected by *Bhlhe40*-KD had similar enrichment in high-glucose-treated cells, too (*Bhlhe40-*siRNA vs Ctrl-siRNA in Glc) (Fig. 2F). Most pathways affected by *Bhlhe40*-KD appear largely independent of ChREBP activation by sugars (Fig. 2F and Fig. S3A, deposited in (18)), cluster around DNA repair and stability, epigenetic regulation, and cell-cycle control (Fig. S3B, deposited in (18)), and may relate to a potential role of BHLHE40 in hepatocellular carcinoma (41). In addition to these pathways, during ChREBP activation by high levels of sugar, *Bhlhe40*-KD caused enrichment of pathways related to inflammation or immune signaling as well as mitochondrial metabolism, oxidative stress, and detoxification processes (Fig. 2F).

### BHLHE40 is necessary and sufficient to activate ChREBP target genes *txnip* and *Rgs16*

**Figure 3:**
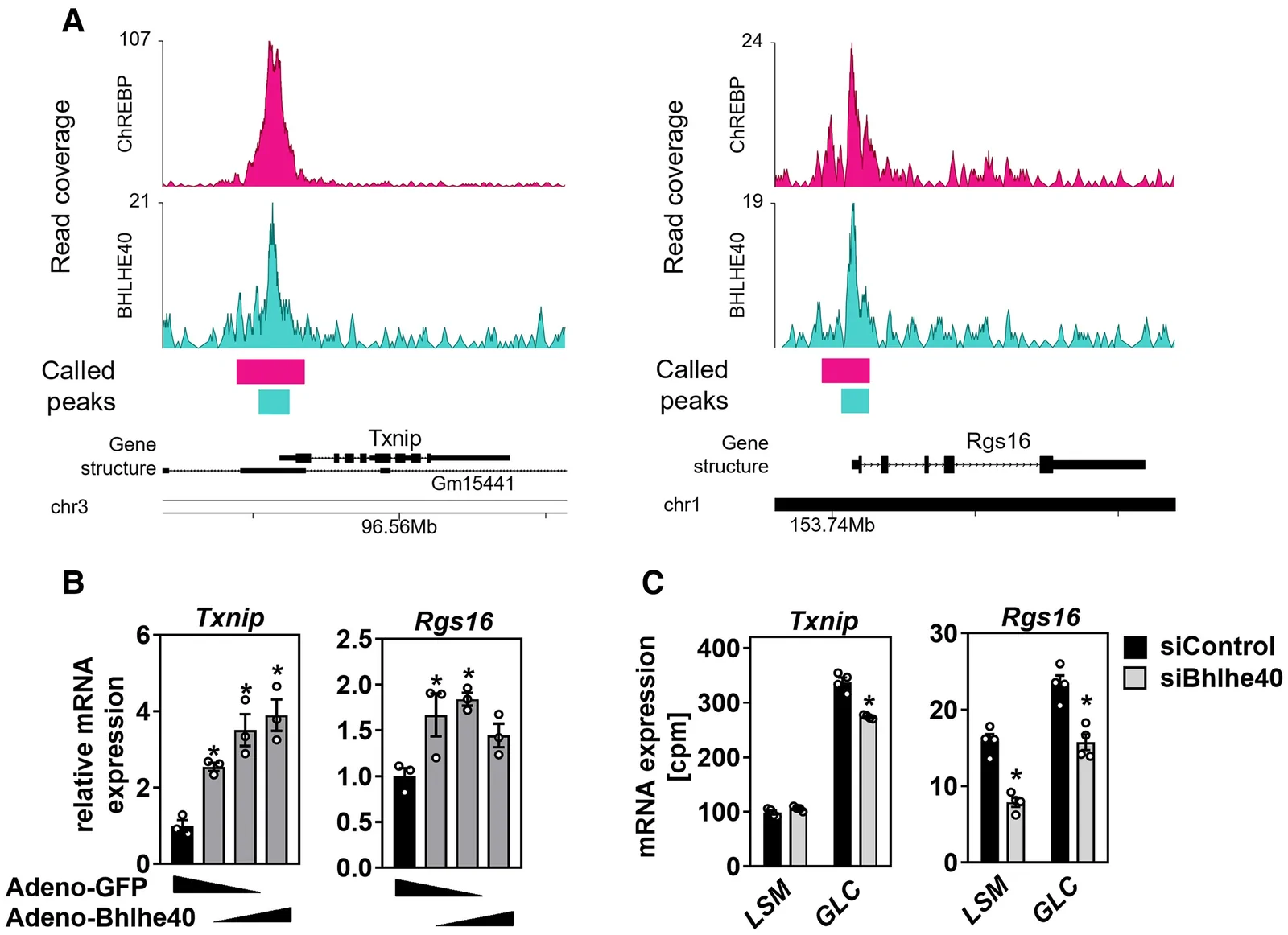
BHLHE40 modulates the expression of ChREBP targets genes. (A) Read coverage for ChREBP and BHLHE40 near the *Txnip* and *Rgs16* genes. (B) *Txnip* mRNA (low Adeno-Bhlhe40 *P = .0180, medium Adeno-Bhlhe40 *P = .0011, high Adeno-Bhlhe40 *P = .0004), and *Rgs16* mRNA *(*low Adeno-Bhlhe40 *P = .0287, medium Adeno-Bhlhe40 *P = .0088) expression levels were analyzed by qPCR in primary hepatocytes with adenoviral overexpression of BHLHE40 and exposed to high-glucose media. (C) *Txnip (*GLC *P = .0003) and Rgs16 (LSM *P = .0003; GLC *P = .0025) mRNA expression levels were analyzed by RNA-Seq in primary hepatocytes depleted of *Bhlhe40* by siRNA in LSM or high glucose (GLC). Data are shown as individual data points and mean ± SEM, *P < .05 vs Adeno-GFP-treated cells or vs siControl by 1-way ANOVA (B) or unpaired 2-tailed Student *t*-test (C). N = 3-4, as indicated by individual data points.

Considering the previous findings on BHLHE40 as a negative feedback regulator of sugar-responsive ChREBP targets *Fasn* and *Pklr* (10), we wanted to test if other well-established ChREBP targets follow the same regulatory principles. We focused on *Txnip* and *Rgs16*, both of which are known to be directly activated by ChREBP (42, 43) and are direct targets of BHLHE40, based on our ChIP-seq data (Fig. 3A). To this end, primary mouse hepatocytes were cultured in high-glucose media to maintain a high ChREBP activation and transduced with adenoviruses expressing GFP (Control) or BHLHE40. Overexpression of BHLHE40 led to a higher expression of both genes, suggesting that these ChREBP target genes can be activated by increased BHLHE40 activity (Fig. 3B). Consistent with being ChREBP targets, the expression of both mRNAs was elevated in primary hepatocytes cultured in high glucose (Fig. 3C). Moreover, depletion of BHLHE40 by siRNA blunted the elevated mRNA levels of both genes, in line with BHLHE40 being a positive regulator of both target genes (Fig. 3C). Thus, our data suggest that, in addition to being a repressor of some shared targets with ChREBP (10), BHLHE40 also has potential to serve as a positive regulator of ChREBP-mediated gene activation. Collectively, our data show that the interplay between BHLHE40 and ChREBP is broader and more complex than previously reported.

### BHLHE40 and ChREBP associate with enhancer clusters containing hepatic nuclear receptors

**Figure 4:**
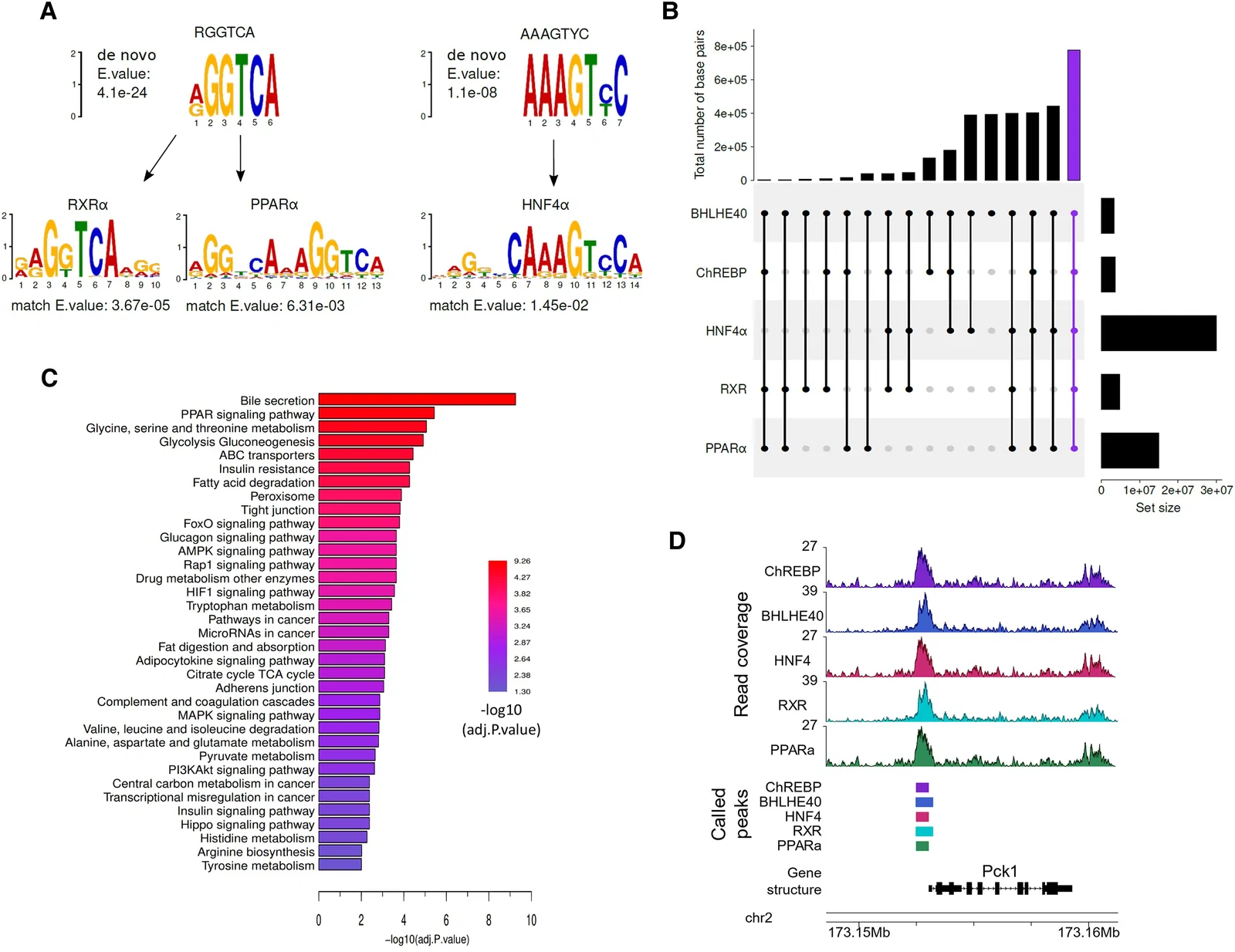
BHLHE40 and ChREBP associate with hepatic nuclear receptors. (A) Binding motif analysis using de novo motif enrichment (DREME) and motif similarity prediction using Tomtom algorithm. (B) Intersection analysis of binding sites from ChIP-seq data for BHLHE40, ChREBP, HNF4Α (GSE90533), RXRα [GSE35262], and PPARα [GSE61817]). (C) Pathway analysis of the genes bound by all 5 transcription factors. (D) Examples of genomic binding near the *Pck1* locus.

Our ChIP-seq and transcriptomics data showed a striking difference in the significantly enriched pathways. This made us hypothesize that BHLHE40 and ChREBP can be functionally complemented by other TFs targeting these genes. To explore this possibility, we performed a motif enrichment analysis for overlapping BHLHE40 and ChREBP ChIP-seq peak areas using motif-based sequence analysis tool MEME-ChIP (v. 4.12.0) (33). Based on de novo motif enrichment program DREME (44), we observed a strong enrichment of motifs “RGGTCA” (E-value = 4.1*10^−24^) and “AAAGTYC” (E-value = 1.1*10^−8^) in the common target genes of BHLHE40 and ChREBP. Using the motif comparison tool Tomtom (v. 5.1.0) (35) we found that these 2 motifs significantly matched the known binding motifs of RXRα (E-value = 3.67*10^−5^), PPARα (E-value = 6.31*10^−3^), and HNF4α (E-value = 1.45*10^−2^) (Fig. 4A).

ChIP-seq datasets for RXRα (GSE35262), PPARα (GSE61817), and HNF4α (GSE90533) in mouse liver were previously reported and are publicly available (19). Comparative analysis of these ChIP-seq datasets with our BHLHE40 and ChREBP data revealed a strikingly high number of shared targets (q-value < 0.05) between the 5 TFs (Fig. 4B). Pathway enrichment analysis (adj. *P* < .05) for the common (5xTF) target genes revealed the potential regulation of many key metabolic processes, including amino acid and central carbon metabolism as well as bile secretion (Fig. 4C). Examples of these common targets include *Pck1*, involved in gluconeogenesis (Fig. 4D), *Gpt2,* involved in alanine metabolism*, Ppargc1α,* involved in mitochondrial biogenesis, and *Adipor2*, a regulator of glucose and lipid metabolism (Fig. S4, deposited in (18)).

### BHLHE40 modulates PPARα target gene expression

**Figure 5:**
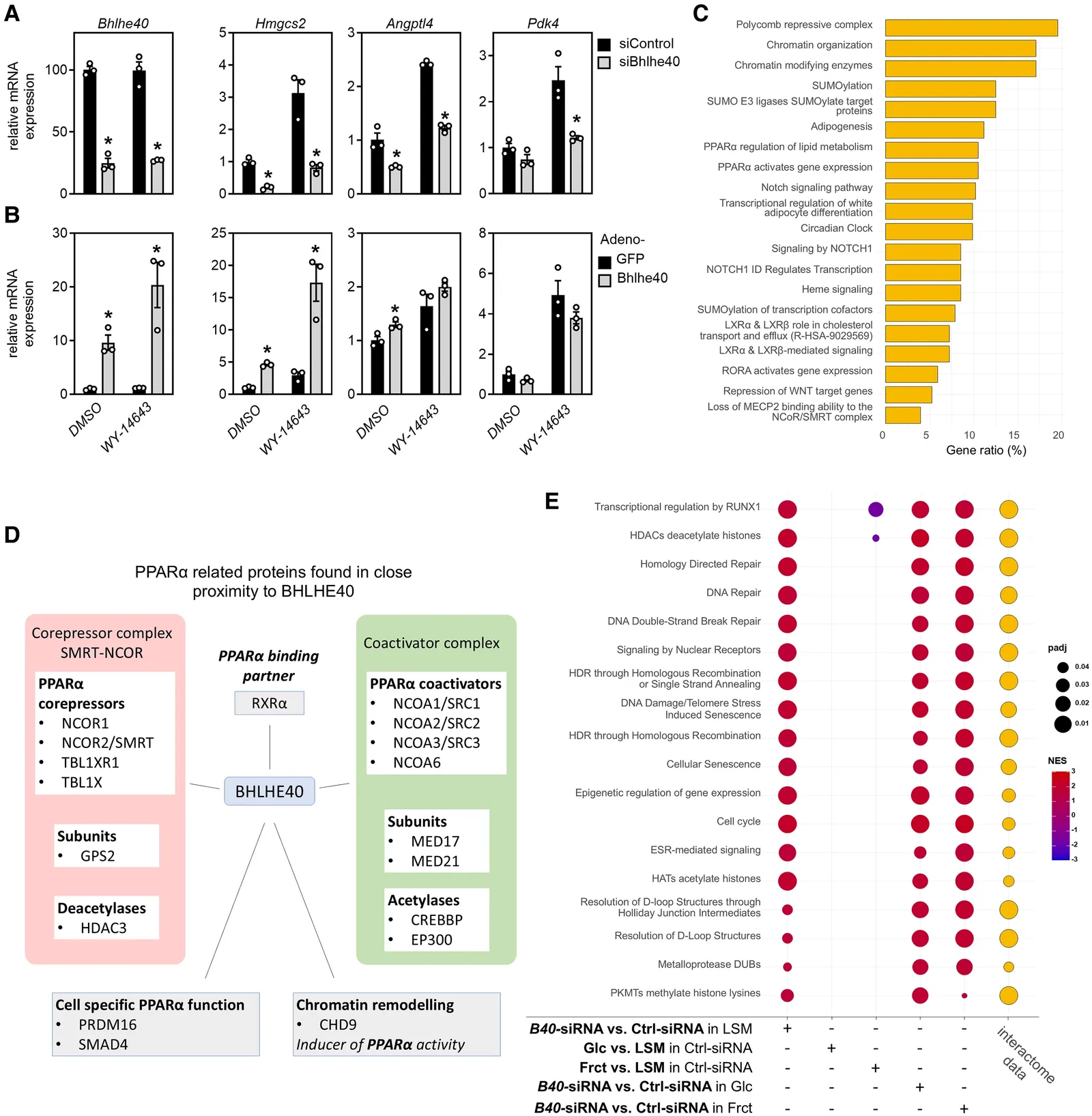
BHLHE40 modulates PPARα activity. Induction of PPARα target genes by its agonist ligand WY-14643 in primary hepatocytes (A) depleted of *Bhlhe40* by siRNA (*Bhlhe40*: DMSO *P < .0001, WY-14643 *P = .0005; *Hmgcs2*: DMSO *P = .0004, WY-14643 *P = .0054; *Angptl4*: DMSO *P = .0153, WY-14643 *P* < .0001; *Pdk4*: WY-14643 *P = .0145) or (B) with adenoviral BHLHE40 overexpression (*Bhlhe40*: DMSO *P < .0038, WY-14643 *P = .0104; *Hmgcs2*: DMSO *P < .0001, WY-14643 *P = .0080; *Angptl4*: DMSO *P = .0269). (C) Overrepresentation analysis of proteins interacting with human BHLHE40 in HEK293 cells. Top 20 pathways (adj. *P* < 10^−8^) sorted according to the adj. *P* value (D) PPARα-related proteins found in close proximity to BHLHE40. (E) Significantly enriched pathways that are common between RNA-Seq (GSEA) and interactome data (ORA). In (A) and (B), data are shown as individual data points and mean ± SEM, *P < .05 vs siControl or vs GFP by unpaired 2-tailed Student *t*-test.

To functionally test the predicted interaction between BHLHE40 and PPARα in modulating gene expression, we assessed the activation of PPARα in primary hepatocytes by the agonist ligand WY-14643 in cells with depletion or overexpression of BHLHE40. The classical PPARα target genes *Hmgcs2*, *Angptl4*, and *Pdk4* were all induced by WY-14643, as expected, whereas depletion of BHLHE40 consistently suppressed this effect (Fig. 5A). Of these genes, only *Hmgcs2* responded strongly to BHLHE40 overexpression (Fig. 5B). These findings are consistent with the model that BHLHE40 facilitates PPARα-mediated gene activation.

To complement these genomic findings, we performed biotin proximity labeling assay to determine the protein interactors of BHLHE40 in stable inducible cell lines generated from HEK293 cells. Despite the limitation of not being of hepatic origin, this cell line provided us with the access to molecular tools necessary for the unbiased proximity labeling analysis (36). Using this approach, we found that BHLHE40 interacts with several proteins related to PPARα-regulated pathways, ranging from coactivators and corepressors to cell-specific transcription factors (Fig. 5C and 5D). In addition, BHLHE40 interacts with RXRα, the obligatory binding partner of PPARα and several other nuclear receptors, with proteins involved in LXR-regulated pathways, and 119 other transcription factors (Fig. 5C, and Supplementary files S1 and S2 deposited in (18)) (45). Overall, the pathway enrichment analysis (adj. *P* < .05) of the interactome data further indicates a broad role of BHLHE40 in transcriptional regulation and chromatin modification (Fig. 5C). Comparison of BHLHE40 specific pathways in mouse hepatocytes (Fig. S4, deposited in (18)) and the interactome pathways in human HEK293 cells (Fig. 5C) also highlight common pathways related to either genome interaction and modification, or gene regulation (Fig. 5E). These results suggest a complex interaction of BHLHE40 with other transcription factors in broadly regulating hepatic gene expression.

## Discussion

Our genomic analyses reveal that BHLHE40 and the glucose-sensing factor ChREBP co-bind many metabolic genes in hepatocytes. BHLHE40 ChIP-seq peaks are enriched near genes in amino acid, TCA cycle, peroxisomal, and AMPK/PPAR signaling pathways, with approximately 54% target-gene overlap between BHLHE40 and ChREBP. Many common targets (eg, *Txnip*) are established ChREBP-responsive genes, consistent with ChREBP's well-known role in driving hepatic lipogenic and glycolytic programs in response to carbohydrate signals (fed state) (46). We find that ChREBP binds directly to the *Bhlhe40* promoter (Fig. 1D and 1E), consistent with prior reports that BHLHE40 is a sugar-induced target of ChREBP (10, 11).

Although *Bhlhe40* mRNA expression in the liver has circadian rhythmicity, with higher levels during the dark period, BHLHE40 target genes in hepatocytes are scarcely known (10, 13). Here, we show that in mouse primary hepatocyte culture, knockdown, or overexpression of BHLHE40 produced concentration-dependent changes in canonical ChREBP targets under high glucose, confirming its impact on specific metabolic genes. Surprisingly, when we analyzed the genome-wide transcriptomic effects of *Bhlhe40* knockdown and BHLHE40 interactome network we observed that many of the enriched pathways are related to DNA repair/stability, cell-cycle control, and epigenetic regulation. These broad effects extend beyond glucose/lipid metabolism and suggest a potential role of BHLHE40 in maintaining genomic integrity and cellular homeostasis, which in hepatocytes may have implications in restraining their oncogenic dedifferentiation (41).

The transcriptomics data showed minimal effect of BHLHE40 depletion on metabolic pathways (Fig. 2F), which is in contrast with our ChIP-seq results that show BHLHE40 binding, together with ChREBP, to regulatory regions of many metabolic genes (Fig. 1H). This discrepancy might be explained by the finding that BHLHE40 and ChREBP co-occupy enhancers together with classical hepatic nuclear receptors, which may compensate for the loss of BHLHE40. De novo motif analysis of overlapping BHLHE40/ChREBP peaks identified enrichment of RGGTCA and AAAGTYC motifs, which match the genomic-binding sites of RXRα, PPARα, and HNF4α. Indeed, comparison with published ChIP-seq datasets shows extensive sharing of genomic targets among BHLHE40, ChREBP, PPARα, RXRα, and HNF4α. This multivalent co-occupancy is in line with the general concept of dense TF clustering (47, 48). In the liver context, these enhancer clusters likely integrate diverse signals—carbohydrate (via ChREBP), lipid (via PPARα/RXR), and developmental/circadian cues (via HNF4, BHLHE40)—to fine-tune gene expression (6). The frequent cobinding of feeding-activated (ChREBP, BHLHE40) and fasting-activated (PPARα/RXR) factors suggests a regulatory nexus where the balance of nutritional inputs is sensed and harmonized (46). This can be further tuned by LXRs nuclear receptors which directly control *Bhlhe40* gene expression to regulate inflammatory and metabolic pathways in the liver (15).

Our genome-wide transcriptomic analysis after *Bhlhe40* knockdown generally supports its role as a transcriptional repressor but also highlights that some ChREBP target genes can be activated by BHLHE40 (7, 40, 49). A previous report showed a negative feedback loop between ChREBP and BHLHE40 regulates the expression of *Fasn* and *Pklr* genes in rat hepatocytes, which is in contrast with our data on *Txnip* and *Rgs16* genes (10). To reconcile these findings, we suggest that the feedback regulation between BHLHE40 and ChREBP is likely fine-tuned by other interacting partners. This is supported by our ChIP-seq analysis in the liver, where BHLHE40 binds many common genes with PPARα, RXRα, and HNF4α. Although limited by the nonhepatic HEK293 cell model used, the unbiased interactome data align with our ChIP-seq data, providing evidence that BHLHE40 can interact with RXRα as well as PPARα coactivators and corepressors.

Together, our data reveal that BHLHE40 is a key component of hepatic enhancer clusters with ChREBP and the core hepatic nuclear receptors PPARα, RXRα, and HNF4α. Understanding how BHLHE40 dynamically engage these enhancers together with other transcription factors, will be important for deciphering liver metabolic regulation and may highlight new avenues for targeting metabolic diseases.

## Data Availability

Original data generated and analyzed during this study are included in this published article or in the data repositories listed in References.

## Abbreviations

BHLHE40: Basic Helix-Loop-Helix Family Member E40
ChIP-seq: chromatin immunoprecipitation sequencing
ChREBP: carbohydrate response-element binding protein
DEG: differentially expressed gene
LSM: low-sugar media
O/N: overnight
qPCR: quantitative PCR
RT: room temperature
TF: transcription factor
